# Association of the European Lactase Persistence Variant (LCT-13910 C>T Polymorphism) with Obesity in the Canary Islands

**DOI:** 10.1371/journal.pone.0043978

**Published:** 2012-08-24

**Authors:** Ricardo Almon, Eva Elisa Álvarez-León, Lluís Serra-Majem

**Affiliations:** 1 Family Medicine Research Centre, School of Health and Medical Sciences, Örebro University, Örebro, Sweden; 2 Preventive Medicine Service, Canary Health Service, Department of Clinical Sciences, Faculty of Health Sciences, University of Las Palmas de Gran Canaria, Canary Islands, Spain; University of Utah, United States of America

## Abstract

**Background:**

European lactose tolerance genotype (LCT -13910 C>T, rs4988234) has been positively associated to body mass indexes (BMI) in a meta-analysis of 31,720 individuals of northern and central European descent. A strong association of lactase persistence (LP) with BMI and obesity has also been traced in a Spanish Mediterranean population. The aim of this study was to analyze a potential association of LP compared to lactase non-persistence (LNP) with BMI in inhabitants of the Canary Islands of Spain using Mendelian randomization.

**Methods:**

A representative, randomly sampled population of adults belonging to the Canary Islands Nutrition Survey (ENCA) in Spain, aged 18–75 years (n = 551), was genotyped for the LCT – 13910 C>T polymorphism. Milk consumption was assessed by a validated questionnaire. Anthropometric variables were directly measured. WHO classification of BMI was used.

**Results:**

LP individuals were significantly more obese than LNP subjects (*χ^2^* = 10.59; *p*<0.005). LP showed in a multivariate linear regression analysis showed a positive association of LP with BMI compared to LNP, (*β* = 0.96; 95% CI: 0.08–1.85, *p = *0.033). In a multinomial logistic regression analysis normal range weight LP subjects showed an odds ratio for obesity of 2.41; 95%CI 1.39–418, (*p* = 0.002) compared to LNP.

**Conclusions:**

The T-13910 of the allele LCT-13910 C>T polymorphism is positively associated with BMI. LP increases significantly the risk to develop obesity in the studied population. The LCT-13910 C>T polymorphism stands proxy for the lifetime exposure pattern, milk intake, that may increase susceptibility to obesity and to obesity related pathologies.

## Introduction

European lactase persistence (LCT-13910 C>T, rs4988234) genotype has been positively associated with BMI in a meta-analysis of 31 720 individuals of northern and central European descent [1]. A strong association of LP with BMI and obesity has been previously reported in a Spanish Mediterranean population [2]. Furthermore, LP has shown to be associated with the metabolic syndrome (MetS) in a population of the Canary Islands in Spain [3]. The Canary Islands are a Spanish region formed by seven islands located in the Atlantic Ocean off the African coast of Morocco. The Canary Islands have about 2 million inhabitants and enjoy a sub-tropical climate.

The aim of this work was to assess if the LCT-13910 C>T polymorphism is associated with BMI given the positive association with MetS already established. Mendelian randomization (MR) is used to explore the relationship of the LCT-13910 polymorphism to BMI. The LCT-13910 C>T polymorphism is a proxy for the lifetime exposure variable of milk intake.

The LCT-13910 C>T single nucleotide polymorphism (SNP) affects the quantity of lactase enzyme produced in the intestinal epithelial cells by interacting with the LCT-gene. The dominant T allele mutation corresponds perfectly with the LP genotype. Heterozygotes are thus considered LP. LNP (lactose intolerance) is given by homozygosis for the C allele rendering LNP an autosomal-recessive trait leading to maldigestion of lactose [4], [5].

LP individuals show an undisrupted lactase enzyme production through their whole life, and are able to consume higher amounts of lactose, and thereby higher amounts of milk and milk products, without symptoms of lactose intolerance compared to LNP individuals [5], [6].

The LCT-13910 C>T SNP genotype is associated with higher milk consumption among individuals of European descent [3], [6]-[10]. This variant has been under strong positive selection. It seems to be related to events of domestic cattle farming (gene-culture co-evolution) [10], [11] and is considered a prime example for human genetic adaptation [11], [12].

In the present study we investigate if LP shows an association with BMI in randomly sampled population of the Canary Islands. This population displays several features worth addressing this question. The Canary Islands exhibit, on account of their history and geographic location, nutritional singularities when compared to the rest of Spain and other Mediterranean countries. The Canary Islands show the highest average milk consumption per capita in Spain, comparable to the milk consumption rates in Nordic countries. Furthermore, the prevalence of obesity is one of the highest here than in all of the Spanish autonomous regions [13]–[16] and finally, the Canary Islands have one of the highest cardiovascular mortality rates in Spain. We have shown in a previous paper that LP increases the risk to develop MetS [3], but it was not clear if this also applies to a higher risk of overweight and obesity.

For that reason, we consider important to analyze this association previously observed in other populations, with the aim to improve the evidence that links polymorphisms and obesity, and particularly taking into consideration the specific characteristics of the Canarian Archipelago.

## Methods

### Population

A representative sample of the Canarian general population, aged 6–75 years, was sampled using a two-stage stratified sampling method from a total of 1,747 individuals who participated in the Canary Islands Nutrition Survey (ENCA). A representative subsample of 782 subjects was randomized to participate in the biochemical assessment. The present study is based on 551 adults aged 18–75 years (240 men and 311 women) with complete genetic data. Anthropometric variables were directly measured. Sociodemographic and lifestyle variables including age, sex, education, smoking, alcohol consumption and physical activity were recorded. Details on data collection have been published elsewhere [14], [17].

### Genetic analysis

For the genetic analysis LP and LNP, genomic DNA was isolated from EDTA whole blood samples from the individuals with the QIAamp DNA Blood Mini Kit spin procedure. The DNA fragment spanning the -13910-C/T polymorphic site was amplified using a biotinylated forward-primer (5′ GGGCTGGCAATACAGATAAGATA-3′) and an unbiotinylated reverse-primer (5′ AGCAGGGCTCAAAGAACAATCTA-3′). The applied sequencing primer was: 5′-CTTTGAGGCCAGGG-3′. Sequencing was performed using a PSQ96 SNP reagent Kit and a PSQ 96MA system (Pyrosequencing AB) PSQ 96MA 2.0.1 software. The procedure has been previously described in detail [18], [19].

### Mendelian Randomization (MR)

Since the carriage of the LCT −13910 C>T polymorphism is subject to random assortment of maternal and paternal alleles at the time of gamete formation associations of LCT genotypes with BMI should not be subject to reverse causality. This is a basic assumption of MR [20], [21]. MR studies modifiable causes of disease in genetic epidemiology. A functional genetic variant, in our study LCT–13910 C>T polymorphism, acts as a proxy for modifiable lifetime exposure patterns (milk consumption). The LCT–13910 C>T polymorphism is known to influence milk consumption (our study's exposure variable) [22]–[26]. According to Mendel's second law of independent assortment, the inheritance of one trait also is independent of the inheritance of other traits. Thus, associations between genetic variants and outcome are not generally confounded by behavioral, physiological or environmental exposures, and observational studies of genetic variants have similar properties to intention to treat analyses in randomized controlled trials [20], [21], [27], [28].

### Statistical analysis

Categorical variables were expressed as frequencies and percentages. Continuous variables were expressed as means and standard deviations. Differences between LP and LNP subjects concerning continuous variables were calculated by Student *t*-test as regards daily energy intake (kcal/d), and with multivariate linear regression analysis models. Adjustments were performed for sex, age, milk avoidance (milk non-consumers vs milk consumers), daily energy intake (kcal/d), educational status (less than elementary, elementary, high school, university) and physical activity (sedentary, light, moderate, vigorous). Differences in the prevalence of normal range, overweight and obesity (according to WHO BMI classification for adults [29] between LP and LNP subjects were calculated by means of χ^2^ analysis and compared using *Z*-test. Differences in the frequency of LP and LNP in the group of milk avoiders were tested by χ^2^ analysis. Student *t*-test was performed with milk intake (g/d) as dependent variable and as LP/LNP independent variable. A stepwise multivariate linear regression analysis was performed with BMI (continuous variable) as a dependent variable and LP/LNP (coded as 1 and 0, respectively) as an independent variable; adjustments were performed for sex, age, milk avoidance, daily energy intake, educational status and physical activity. Ordinal regression showed lack of parallelism and the null hypothesis of parallel lines was rejected, and a multinomial logistic regression was used to assess the predictive value of LP/LNP as regards development of obesity as dependent outcome. Lactase persistence (LCT-13910 genotypes: CT/TT, LP) and Lactase non-persistence (LCT-13910 genotype: CC, LNP), sex and age group were used as factors. Covariates included in the model were daily energy intake, daily milk intake, milk avoidance, educational status and physical activity. Statistical significance was set at 0.05, and confidence intervals at 95%. All analyses were conducted using SPSS for Windows (version 19.0; SPSS Chicago, IL, USA).

### Ethics

All study patients submitted informed, written consent forms.

## Results

Genotyping for the LCT-13910 C>T polymorphism was performed in 551 adult individuals aged 18 to 75 years (mean age was 45.5 years); 311 (56.4%) were women and 240 (43.6%) were men. Seventy-two (13.1%) subjects were homozygous for LP (TT), 258 (46.8%) were heterozygotes (CT) and 221 (40.1%) were homozygous for LNP (CC). Genotype frequencies were consistent with Hardy-Weinberg equilibrium (HWE), (*χ^2^* = 0.059; *p* = 0.807). The T-13910 allele was present in 59.9% (n = 330) of the genotyped population, rendering these individuals LP. LNP was present in 40.1% (n = 221). Forty-eight subjects were milk-avoiders (LP: n = 29/330, LNP: n = 19/221, *χ^2^* = 0.006; *p* = 0.938).

BMI data were available for 526 individuals. In 10 cases data for height (cm) was not present, in 5 cases weight (kg) was not present. In 10 individuals both data for height and BMI were missing (missing: n = 25).

**Figure 1:**
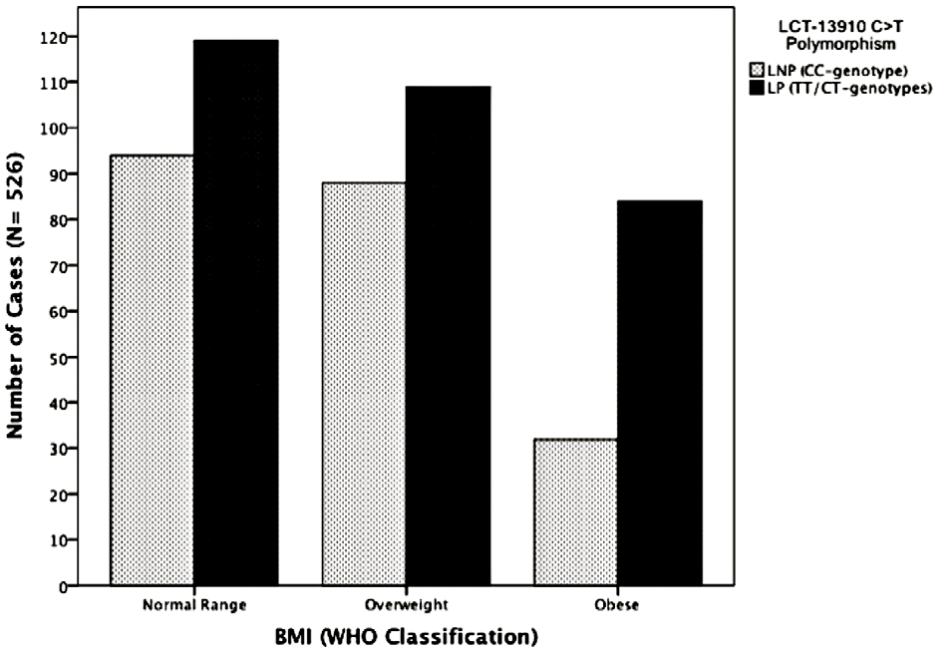
BMI classification by LCT genotypes (LP: n = 330; LNP: n = 221) N = 13 individuals were underweight (n = 11 showed mild thinness and n = 2 moderate thinness; no subject showed severe thinness). These subjects were included in the normal range column. Normal Range: LP = 119; LNP = 94, overweight: LP = 109; LNP = 88, obesity: LP = 84; LNP = 32; Missing BMI in 25 cases (N = 526).

WHO classification for BMI was used [29]. Normal range (including mild and moderate thinness) was found in 213 subjects (40.5%), 197 (37.5%) subjects were overweight and 116 (22.1%) were obese. Mild and moderate thinness was present in 13 subjects (mild thinness in 11 cases, and 2 individuals showed moderate thinness), (Figure 1).

Individuals were grouped by age: 18–39 years, 40–59 years and 60–75 years [30]. Daily energy intake did not significantly differ between LP and LNP subjects. LP subjects consumed on average 1,670 kcal/day and LNP subjects consumed on average 1,690 kcal/day (Table 1).

**Table 1:** Population characteristics by LCT-13910 genotypes.

Characteristic	Subcharacteristic	TT (n = 72)	CT (n = 258)	CC (n = 221)
Age, years		46.1 (14.2)	45.9 (15.0)	44.8 (15.0)
Sex (%)				
	Women	44 (14.1)	148 (47.6)	119 (38.3)
	Men	28 (11.7)	110 (45.8)	102 (42.5)
Body mass index (BMI), (kg/m^2^)		26.5 (4.5)	27.2 (5.7)	26.1 (5.2)
BMI (WHO-Classification) (%)				
	Normal range (18.50–24.99)	25 (37.3)	94 (38.2)	96 (44.4)
	Overweight (25.00–29.99)	27 (40.3)	83 (33.7)	88 (40.7)
	Obese (>30.00)	15 (22.4)	69 (28.1)	32 (14.9)
Energy intake, kcal/day		1614 (339)	1680 (351)	1691 (349)
Milk intake, g/day^1^		295 (152)	302 (211)	246 (168)
Physical activity (%)				
	Sedentary	44 (62.9)	169 (66.5)	137 (62.6)
	Light	20 (28.6)	59 (23.2)	59 (26.9)
	Moderate	5 (7.1)	22 (8.7)	19 (8.7)
	Vigorous	1 (1.4)	4 (1.6)	4 (1.8)
Education (%)				
	Less then elementary	24 (33.8)	89 (34.5)	76 (34.7)
	Elementary	21 (29.6)	75 (29.1)	52 (23.7)
	High School	18 (25.4)	56 (21.7)	65 (29.7)
	University	8 (11.2)	38 (14.7)	26 (11.9)

1 *p* = 0.001 (LCT-13910 CC vs LCT-13910 CT/TT); Student *t*-test (36 missing because of incomplete data).

Table 1 shows the population characteristics by genotype. LP subjects consumed significantly more milk than LNP subjects, 300 g/day average intakes by LP subjects and 246 g/day by LNP subjects (*p = *0.001; Student *t*-test) (Table 1).

LP individuals were significantly more obese than LNP subjects (LP: 16%, LNP: 6%; *χ^2^* = 10, 59; *p = *0.005), (Figure 1). *Z*-test for proportions showed a statistically significant difference at the 0.05 level between LP and LNP as regards the prevalence of obesity. Overweight and normal range classifications of BMI did not differ significantly between LP and LNP.

A stepwise multivariate linear analysis was performed with BMI as a dependent variable. Adjustments were performed for sex, age, milk avoidance, daily energy intake, educational status and physical activity. A positive association of LP with BMI, compared to LNP, was found. In model 1 adjustments for age, sex, energy intake and milk intake were performed (*β* = 0.95; 95% CI: 0.06–1.83; *p = *0.037, *R* ^2^ = 0.12). In model 2 further adjustments for milk avoiders, physical activity and educational status were included (*β* = 0.96; 95% CI: 0.08–1.85; *p = *0.033, *R* ^2^ = 0.16), (Table 2).

**Table 2:** Associations of LP with BMI (continuous outcome) compared to LNP in a multivariate linear regression analysis.

Variable	Model 1^1^			Model 1^2^		
LCT-13910 C>T (LP/LNP)	*β* (95%CI)	*p*-value	R^2^	*β* (95%CI)	*p*-value	R^2^
LP (Reference LNP)	0.95 (0.06–1.83)	0.037	00.12	0.96 (0.08–1.85)	0.033	00.16

1 Adjusted for age, sex, energy intake, milk intake.

2 Adjusted for model 1 plus milk avoidance, physical activity, educational status (36 missing because of incomplete data).

A multinomial logistic regression model was used to assess the Odds Ratio (OR) of LP for obesity. WHO classification of BMI was used as an outcome variable, (Figure 1). Lactase persistence (LCT-13910 genotypes: CT/TT) and Lactase non-persistence (LCT-13910 genotype: CC), sex and age group were factors. Covariates included in the model were daily energy intake, daily milk intake, milk avoiders, educational status and physical activity. LP subjects having normal range BMI showed an OR to develop obesity of 2.41; 95%CI 1.39–4.18; (*p* = 0.002) and overweight LP subjects showed an OR of 2.32; 95%CI 1.39–3.88; (*p* = 0.001) to develop obesity, (Table 3).

**Table 3:** Estimated Odds Ratios and 95% Intervals for Obesity Relative to Normal Range weight and Overweight^1^.

Dependent variable: Body mass index (WHO classification)	Predictor (LCT-13910C>T)	Odds Ratio	95% CI	*p*-value
Normal range (BMI: 18.50–24.99) (Reference:Obese)	LP (Reference: LNP)	2.41	1.39–4.18	0.002
Overweight (BMI: 25.00–29.99) (Reference:Obese)	LP (Reference: LNP)	2.32	1.39–3.88	0.001

1 Adjusted for sex, age, energy intake, milk intake, milk avoidance, physical activity and educational status (36 missing because of incomplete data).

## Discussion

Lactose tolerant (LP) individuals from the Canary Islands show a more than 2 fold higher risk to develop obesity than lactose intolerant (LNP) subjects. The LP variant might have comparable or potentially even a higher effect size as the ‘fat mass and obesity associated gene (FTO)’ in some European populations [1]. Surprisingly, the association of LP with BMI has remained mainly undetected by genome-wide association studies (GWAS) [1]. The first report based on GWAS of a possible association of LP with BMI was that of Meyre et al. [31].

In an earlier study, performed on the same representative sample of the general population of the Canary Islands, we found a 57% higher risk of LP subjects to develop metabolic syndrome (MetS) compared to LNP subjects [3]. We believe that this observation might have been mediated to some extent by the effect LP has on obesity. Hence, LP not only could display an effect on obesity, but also to obesity related pathologies like MetS [3] and potentially cardiovascular disease (CVD).

A notable north-south gradient, as regards both prevalence and incidence of CVD [32] and the prevalence of LP [33], can be found across Europe. Even though the Canary Islands, the most southern of all Spanish autonomous regions, show the highest average milk and dairy consumption in Spain. The per capita amount of ingested milk is comparable to the average milk intakes in Nordic countries [15]. At the same time Canary Islands population displays a high prevalence of cardiovascular risk factors and one of the highest cardiovascular mortality rates in Spain. However, the role that milk consumption plays in the development of CVD is controversial [34], [35], and it keeps to be elucidated, if LP might or might not be involved in intermediate phenotypes of CVD. Furthermore the question, if the LP variant has multiple biologic effects, also remains to be clarified [36].

MR has been used in this study. The main assumption in this study is that LP (lactose tolerance) individuals consume in average significantly more milk than LNP (lactose intolerance) individuals throughout their lifetime, and not only at the moment dietary intakes were assessed. If this assumption is correct, the LCT-13910 C>T polymorphism can be used as a proxy measure for lifetime exposure to milk intake patterns. Cultural influences on milk consumption might be able to override the discomforts consequent on milk ingestion in lactose intolerant individuals. This, nevertheless, could not be observed in our sample of the general population of the Canary Islands. Neither have we been able to observe this in Sweden where dairy product consumption is very common and average milk intake is globally one of the highest. In both samples, Sweden, Canary Islands, LP subjects consumed statistically significantly more milk compared to LNP subjects [8], [13]. A recent study performed on elderly Spanish subjects from the Iberian Peninsula showed a weaker association of LP/LNP genotypes with average dairy consumption and BMI compared to the association we found in the present work [2]. We believe that in this group of elderly individuals dairy consumption might have been conditioned by age and the circumstance that all subjects belonged to a high cardiovascular risk cohort. Furthermore, Canary Islanders exhibit, on account of their history and geographic location, singularities, which also translate in a substantially higher Northwest African genetic influence compared to populations from the Iberian Peninsula [37]. In addition, the Canary Islands show the highest average milk consumption per capita in Spain, comparable to the milk consumption rates in Nordic countries. Taken together these factors may explain in part the stronger association of LP/LNP with milk intake and BMI we found in this work, compared to the study performed on individuals from the Iberian Peninsula.

Potential mechanisms by which the LP variant might exert an effect on body composition are: the more restricted diets as regards milk and dairy intake of LNP compared to LP subjects, differences in gut microbiota that may influence caloric extraction of ingested food between LNP and LP [26], [38], [39] or hormonal/peptides/fatty acids constituents in milk having biologic effects on consumers [40]–[42].

The sample size of this study and possibly the restriction to liquid dairy (milk consumption) may be considered limitations. Nevertheless, there is a strong correlation between liquid dairy (milk) intake and the LP variant [7], [43]. Although we have controlled for the main confounders known to influence body composition, it is possible that other unmeasured confounders, e.g. early life programming, ethnic affiliation or population stratification, breastfed or non-breast fed, biological and/or statistical gene-diet interactions [44] may play a role. We used a candidate gene approach in the present study, but also other genes or variants, for which we have not genotyped in this sample are known to influence BMI, among others: FTO, Insulin induced gene 2 (INSIG2-gene) [45], Melanocortin 4 receptor gene (MC4R gene) [46] or ectonucleotide pyrophosphatase/phosphodiesterase 1 gene (ENPP 1 gene) [47].

We conclude that the European variant behind LP appears to contribute to obesity in the studied population, reinforcing the evidence coming from recent previous studies [1], [2]. The T-allele of the LCT-13910 C>T has been subject to a strong positive selection in recent history enabling an unrestricted diet concerning milk and milk products [10]. In nutritionally replete countries with high average life expectancy, and ad-lib availability of milk and milk products, LP status might increase the susceptibility to develop obesity and to obesity related pathologies.
